# Challenges experienced by community health workers and their motivation to attend a self-management programme

**DOI:** 10.4102/phcfm.v14i1.2911

**Published:** 2022-01-12

**Authors:** Levona J. Johnson, Laura H. Schopp, Firdouza Waggie, José M. Frantz

**Affiliations:** 1 Department of Physiotherapy, Faculty of Community and Health Sciences, University of the Western Cape, Bellville, South Africa; 2 Department of Health Psychology, University of Missouri, Columbia, United States of America; 3 Department of Clinical and Community Engagement, Faculty of Community and Health Sciences, University of the Western Cape, Bellville, South Africa

**Keywords:** challenges, community health workers, health behaviours, motivation, self-management

## Abstract

**Background:**

Community health workers (CHWs) are change agents expected to assist in decreasing the global burden of disease in the communities they serve. However, they themselves have health risk behaviours, which predispose them to non-communicable diseases and thus need to be empowered to make better health choices. There is a gap in literature detailing the challenges faced by CHWs in addressing their own health risk behaviours.

**Aim:**

This study aimed to explore the challenges experienced by CHWs in carrying out their daily duties and the motivating factors to join a self-management programme.

**Setting:**

The study was conducted in a low socio-economic urban area of the Western Cape, South Africa.

**Methods:**

This study used a qualitative exploratory design using in-depth interviews to obtain rich data about the personal and professional challenges that CHWs experience on a daily basis.

**Results:**

Five themes emerged with regard to professional challenges (social conditions, mental health of patients, work environment, patient adherence and communication). This cadre identified ineffective self-management as a personal challenge and two themes emerged as motivation for participating in a self-management programme: empowerment and widening perspective.

**Conclusion:**

The challenges raised by the CHWs have a direct impact on their role in communities. This study therefore highlights an urgent need for policymakers and leaders who plan training programmes to take intentional strategic action to address their health challenges and to consider utilising a self-management intervention model to improve their overall health status.

## Introduction

Community health workers (CHWs) are central to driving health promotion and prevention strategies as part of community-based primary healthcare infrastructure. The global shortage of health professionals has driven an increase in the use of CHWs as a key cadre to meet the health needs of society. Globally, there is a shortage of health workers such as midwives, nurses and physicians.^1^ Parallel to this shortage in the workforce is the increase in the disease burden in resource-constrained countries with an increase in communicable and non-communicable diseases.^2^ Sub-Saharan Africa has only 3% of the total health workers in the world and this group provides service to a region that carries 24% of the global burden of disease.^3^ South Africa has an inequitable distribution of medical resources as a result of the apartheid epoch, and has struggled to increase the formal health force with skilled health professionals, as many health workers opt to relocate to countries that provide higher incomes.^1^ Consequently, the global shortage of health professionals has affirmed the resolve to invest in training more CHWs, to address these health and development needs.^4^ Community health workers are regarded as an integral part of the model designed to increase the number of persons able to meet the health needs of society and simultaneously provide a vehicle for sustainable improvement in global health.^5^

Community health workers have very basic-level healthcare skills and they require training to achieve ‘structural competency’ in primary healthcare practice.^6^ Training helps CHWs to gain understanding of underlying complex structural, disease and individual factors related to patient non-adherence to recommended medical regimens, rather than attributing treatment failures solely to poor patient-level motivation.^7,8^ Structural competence has been defined as ‘the capacity for health professionals to recognise and respond to health and illness as the downstream effects of broad social, political and economic structures.^6^ Community health workers who by definition are trusted members of the communities they serve^9^ understand and are knowledgeable about the environmental, social and political factors and the interpersonal networks community members encounter.^8^ Community health workers’ intimate knowledge of these social determinants, coupled with their role as community advocates, enable CHWs to play a vital two-prong role: (1) to contribute to policy change^10^ to redress imbalances in health care and (2) to foster long-term well-being of communities.^11^ Community health workers have first-hand knowledge and information about local structural factors impeding community health position. They play an ideal role to use this cultural competence to deliver improved patient-centred health services.^12^

The impact of CHWs in the care of non-communicable diseases has garnered empirical support. Community health workers’ roles as health educators, advisors, rehabilitation workers and group support facilitators stand them in good stead to influence community health.^13^ Community health workers also play an important role in helping communities to meet their health and social needs.^14^ As key frontline health workers who are active in communities and have a deep understanding of community needs, they are expected to provide appropriate health information and to advocate on behalf of the neighbourhoods they serve.

Literature highlights that CHWs use their personal experiences to play a crucial role as a buffer between communities and the health system.^15^ A recent study emphasised that it is important that health trainers such as CHWs negotiate the tension between knowledge of health risk behaviours and individual lifestyle choices.^16^ They are considered pivotal players in managing health risk factors and so must serve as local role models. In a study conducted in the African context, which focused on personal physical activity and patient counselling practices, the authors highlighted the need for the credibility of health professionals regarding health education that is in line with their own personal practices.^17^ This is supported by researchers who highlighted the need for health workers to be more proactive in terms of their own risk behaviours and increase their own understanding as to why individuals make certain decisions about health behaviours.^18^

One tool that has been identified as an effective way to promote behaviour change is self-management. Self-management is a health behaviour change strategy,^19^ which zeroes in on the patient’s perception^20^ and allows individuals to enhance their own health status by achieving graduated success in self-determined health goals.^21^ It has been used successfully in a variety of disease management and positive changes in health behaviours were recorded.^22,23^ A recent study^24^ found that CHWs wanted to participate in self-management interventions in order to gain knowledge and skills to help themselves and others. This is important, as CHWs are seen as public health workers who can build individual capacity in communities. This article explored the challenges experienced by CHWs in carrying out their duties and their motivation to join a self-management programme.

## Methods

### Study design

This study employed a qualitative exploratory design using in-depth interviews amongst CHWs prior to enrolment in a self-management programme. The interviews were conducted with two primary purposes, namely to describe the CHWs’ personal and professional challenges in performing their duties and to examine motivations for wanting to join a self-management programme. This study design allows for the in-depth, rich and holistic understanding of the phenomena under investigation.^25^

### Research setting

The study was conducted in an urban setting in the Western Cape, South Africa. According to the 2011 Census of Cape Town, the high rates of poverty (47%) within the community tend to compromise the mental health and well-being of the residents.^26^ The CHWs in this area work in environments, which are both socio-economically poor and have high rates of violence. Approximately, 84% of attempted murders in this area are attributed to gang activity and this region is in the top 10 for illegally possessing firearms and ammunition.^27^

With the re-engineering of primary healthcare in South Africa, the support for the CHW workforce has expanded, consequently they are being employed by non-governmental organisations (NGOs) sanctioned by the provincial health departments.^28^ Those lay healthcare workers who did not have formal training, worked in nurse-led teams^13^ and their focus was directed towards health prevention and promotion.

### Sampling

Participants formed part of a bigger study where they completed a survey related to determining their risk factors for non-communicable diseases.^29^ Based on the survey, participants were purposively selected to be interviewed. Purposive sampling is a technique used in qualitative research to obtain rich information from key participants^30^, thus allowing for recruitment of participants who met the predetermined criteria aligned with study objectives.^30^ Stratified purposive sampling allows the researcher to capture variations in thoughts rather than identify a common core although the latter may emerge during analysis.^30^ Thus, the number of participants is not important, but rather the information obtained from participants. The two inclusion criteria for participation in the study were that CHWs had to be employed as CHWs at the time of the study, and they had to indicate that they were willing to participate in a self-management programme. Participants were excluded if they were not willing to participate in the self-management programme. Fifteen participants were recruited from two NGOs that employ CHWs in the Western Cape.

### Data collection

Data were collected by means of in-depth interviews. In-depth interviews are relevant when comprehensive insight about participants’ concepts, perspectives, behaviours and experiences regarding a specific intervention, idea or situation is sought.^31^ Interviews allow the researcher to understand how participants view the phenomena under investigation.^31,32^ This interview strategy provides a comfortable and natural atmosphere, which facilitates conversation,^32^ as meeting one on one with the interviewer enables those who would normally shy away from contributing opinions in a group setting to voice their perspectives and provides more explicit information than surveys.^31^ In this study interviews were conducted by a trained researcher at a mutually suitable date and venue.^24^ The researcher has a background in physiotherapy and has conducted several interviews as part of research projects. She was also guided by experts as supervisors for this project. Informed consent and permission to record the interviews was obtained prior to interviews, and interviews lasted approximately 20 min – 30 min. Interviews were started with important questions and probes were used during the interview process. The interviews were based around two central questions: ‘What are the challenges you experience personally and professionally in conducting your duties? Why would you be interested to join a self-management programme or what would be your motivation to join a self-management programme?’ Individual interviews were conducted until saturation was achieved. The participants were interviewed in the language they preferred (English/Afrikaans) as the researcher was versatile in both languages. Field notes and observational cues were recorded by the researcher at the end of each interview.

### Data analysis

Each interview was transcribed verbatim. Given the exploratory nature of the study, thematic analysis was conducted following a rich and detailed account from the participants.^33^ The interviews were transcribed verbatim and each transcript read several times by the primary researcher. Initial codes were created by writing notes on the transcripts. Codes were then group and clustered together into agreed upon themes by the researcher and co-researchers.

To promote confidence in the research and ensure quality it is essential that the protocols and procedures employed in the study are detailed.^34^ The following procedures were conducted to ensure trustworthiness: during interviewing the researcher repeated the central questions in different ways to ensure that the participants understood the questions (credibility). In addition, detailed notes were taken during the interview process and interviews were recorded (dependability). Co-researchers who were academics and had expertise in the field of qualitative research, reviewed all notes made during the analysis process and discussed the conclusions reached (confirmability). During the reporting of the findings, actual quotes are used relevant to the participants and a full description of the context is provided (transferability).

### Ethical considerations

Ethical clearance was obtained from the Human and Social Sciences Research Ethics Committee at the University of the Western Cape (HS17/8/23), and permission was obtained from NGOs in the sub-district. Written informed consent was obtained from all respondents and confidentiality was ensured.

## Results

Fifteen participants were interviewed. As indicated in Table 1, CHW participants lived in the communities in which they worked for an average of 21.5 years and have been working as CHWs for an average of 3.7 years. The participants were primarily female (80%), 33% were married and had a mean age of 43.2 years. All the participants received a monthly stipend through the two NGOs. Participants were primarily engaged in home visits, providing home-based assistance with activities of daily living, ensuring adherence to tuberculosis (TB) and human immunodeficiency virus (HIV) medication regimens and conducting blood pressure and blood glucose monitoring.

**TABLE 1:** Description of the participants.

Number	Years living in the community	Years as a community health worker	Age	Type of work	Family
1	37	7	37	Follow up on TB and HIV patients	Single mother of three kids
2	20	5	31	Seniors BP monitoring, exercise and health talks	Single mother with one child
3	4	1	50	Assist elderly patients with ADL	Single mother with two children
4	12	6	42	Assist patients with ADL and at senior clubs do BP readings and sugar testing	Married mother with two children
5	23	7	23	Follow up on TB and HIV patients and breast-feeding counselling	Single mother with two children
6	10	3	46	Home-based care and TB and HIV follow ups	Widowed mother with one child.
7	20	5	52	Home visits and follow up on HIV patients	Divorced mother of three and grandmother of 5 children
8	11	4	42	Work with patients who have TB, HIV and diabetes	Single female
9	24	3	54	Follow up and home visits of HIV patients	Divorced mother of three and grandmother of three
10	7	5	39	Adherence support for TB and HIV patients	Married mother with two children
11	8	2	67	Home assessments (BP etc.)	Married father with children
12	56	1	56	Home visits, massage, medication	Married mother with 8 children and grandmother of 6
13	27	7	43	Manage CHWs	Single mother with one child
14	34	4	34	TB and HIV care	Single male without children
15	30	18	32	TB and HIV care and administration	Married father with two children

CHW, community health workers; TB, tuberculosis; HIV, human immunodeficiency virus; BP, blood pressure; ADL, activities of daily living.

Interpretation of the results yielded five themes (social conditions, mental health of patients, work environment, patient adherence and communication) of challenges experienced by the CHWs in performing their duties. Personal challenges revealed ineffective self-management as a theme, and motivation for participating in a self-management programme was characterised by two themes: CHW empowerment and widening CHWs’ perspectives (Table 2).

**TABLE 2:** Emerging themes of challenges.

Question	Theme	Codes
What are some of the challenges/difficulties you face in: Promoting the health of your patients?	Social conditions	Social circumstances and depression Challenges in the home and understanding fearful behaviour
	Mental health status of patients	Depression in patients Denial Empathy shown by CHWs
	Patient adherence	Lack of patient compliance Lack of understanding
	Work environment	Workload and expectations of the CHWs’ employers
	Communication	Perceived rudeness of patients Patient interaction Stigma associated with certain illnesses, for example, TB and HIV Lack of education in community members
What are some of the challenges/difficulties you face in: Promoting your own health?	Ineffective self-management	Lack of self-care Lack of motivation Personal personality challenges
What would motivate you to participate in a self-management programme?	Empowerment	Get to know myself better Goal oriented
	Widening perspective	Learn new things Improved outlook and understanding

CHW, community health workers; TB, tuberculosis; HIV, human immunodeficiency virus.

Quotations are used to illustrate how the information is rooted in the participants’ perceptions and experiences.

### Challenges experienced by community health workers

The challenges experienced by the CHWs included influence of social conditions, mental health status of the patient, patient adherence, communication and work environment.

#### Social conditions

This theme comprised social factors influencing patient health status, such as the impact of socio-economic status on individual and community mental health:

‘Sometimes it is difficult for us to get through to them in a sense like they have their own house problems as well.’ (Participant 5, female, 23 years old)

Participants also observed that poverty affected patients’ response to healthcare interventions:

‘[*I*]t’s already poor communities so all that circumstances depress these people.’ (Participant 1, female, 37 years old)

The participants also commented about gaining access to patients’ homes and the patients’ reactions that arriving at their homes could evoke. These reactions are because of the CHWs daily interaction in the community and living in fear. They expressed being received poorly by patients, such as instances in which patients rudely addressed CHWs and were dismissive of CHWs when CHWs requested access to patient homes. These CHWs observed that this was particularly prevalent when the CHWs started working in a particular area and were not yet well known to community members.

Community health workers in the study also reported being denied access to clients’ homes, albeit for different reasons. Community health workers observed that clients were concerned that the police were at their door. This is consistent with the high-crime neighbourhoods in which the CHWs conduct their work and police visits were not associated with favourable news or outcomes:

‘I discovered that when you get to people, sometimes … A lot of them are very edgy if you knock they think it is the police.’ (Participant 11, male, 67 years old)

#### Mental health status of patients

This theme captured how participants experienced their patients when doing home visits. Although the CHWs realised that they cannot diagnose patients, they were able to recognise mental health symptoms and demonstrate empathy as they dealt with patients:

‘[*S*]ome of them are very depressed, they have other issues as well, so when you try and speak to them, they are very closed.’ (Participant 1, female, 37 years old)

‘[*M*]ost of them are in denial mode or they don’t want to know they are sick.’ (Participant 2, female, 31 years old)

‘It is basically trying to like bring yourself on the same level as the person because obviously you are not as sick as that person is, so sometimes it is difficult for you to get through to the patient.’ (Participant 5, female, 23 years old)

#### Patient adherence

Community health workers communicated frustration in getting community members to change their health behaviours:

‘She does nothing that I tell her – no exercise, nothing. Every time when I must go to treat her then her skin is everywhere and arms are stiff like rocks, then I massage it right again.’ (Participant 3, female, 50 years old)

Participants expressed a perception that patients refuse to adhere to health advice despite concerted CHW efforts:

‘I struggled a lot to get through because it puts a strain on me especially when I communicated with a patient but they don’t want to listen.’ (Participant 8, female, 42 years old)

Follow up of patients who defaulted treatments can be complicated and unsuccessful:

‘[*W*]hen the patient defaults and you are supposed to go and look for the patient and you find out that this patient doesn’t live there. This patient gives the incorrect address and stuff like that.’ (Participant 13, female, 43 years old)

#### Work environment

Community health workers in the study also identified this struggle to manage workload in their high-need, low-resource community settings:

‘Sometimes it feels like they didn’t think this through because there is just so much that a person can do in a day but then they expect you must have a workload of so much.’ (Participant 1, female, 37 years old)

Occupational stress triggered by perceived inadequate staffing to ensure patient safety was also a factor:

‘I was so scared that I will do something with the old people, because if they fall, I will get into trouble.’ (Participant 3, female, 50 years old)

#### Communication

Participants reported communication challenges when met with disrespect from community members and how they manage this dynamic as they ensure appropriate health service delivery:

‘Some of the patients are very rude. What we came to learn is that one must just be quiet.’ (Participant 9, female, 54 years old)

‘A lot of times I have been in peoples’ homes who are a bit rude, [*previously*] some of the patients were not rude, but they are now so rude.’ (Participant 12, female, 56 years old)

Community health worker participants observed that community members’ lack of education is a factor limiting their ability to comprehend healthcare instructions. Community health workers in this study showed insight into this barrier and were able to utilise their knowledge of the community to advance the treatment or programmes they were administering:

‘[*S*]ometimes we have to explain more detail for the patient because sometimes the people don’t know exactly about TB and then the stigma.’ (Participant 10, female, 39 years old)

‘I mean, not everybody is educated and etcetera, so you have to come down on a level of understanding so that would have to be patience and understanding also.’ (Participant 15, male, 32 years old)

### Challenges with their own health

The second question of this study focused on the challenges that CHWs experienced with their own health. The themes that emerged were ineffective self-management with lack of self-care, lack of motivation and CHWs’ own personality challenges.

#### Ineffective self-management

‘The challenges that I had is to eat healthy and even if I’m on medication and not taking medication.’ (Participant 10, female, 39 years old)

‘For myself, I will say, yes because I’m very lazy hey – I’m not lazy, it’s almost like, I always need a coupling [*colleague*] to do something.’ (Participant 13, female, 43 years old)

‘I faced a lot of challenges and so with that I had destructive ways about me because that was the way I managed things to keep it there and not let it go any further.’ (Participant 15, female, 32 years old)

### Motivations to participate in a self-management programme

The third line of inquiry in interviews focused on why the CHWs would want to engage in a self-management programme. The two themes that emerged were empowerment and widening perspectives.

#### Empowerment

Participants indicated a desire to become more confident and to take control of their own lives, including the need to focus on themselves at times rather than on the community:

‘So obviously that has to do with me personally and I wanted to like, basically just get to know myself much better also than what I do at the moment and manage myself properly also in a way that is pleasing and also pleasant for other people around me.’ *(Participant 15, male, 32 years old)*

‘The main emphasis … – I would say it was like mostly goal orientated and that was, kind of, exactly what I needed. To be honest with you, the way I grew up I wasn’t really motivated and I needed to get motivated.’ (Participant 14, male, 34 years old)

‘For me, it was that I wanted to learn more about myself and when I learn about myself, I can teach other people what I had learnt.’ (Participant 8, female, 42 years old)

#### Widening perspectives

Community health worker participants communicated their interest in gaining new knowledge. New knowledge acquisition fuels possible job advancement (personal benefit) and the transference of new-found knowledge to benefit others (community benefit):

‘I like to learn new things. I like to do so because to me, if I learn new things, tomorrow I come somewhere and I can educate somebody else.’ (Participant 13, female, 43 years old)

‘I told myself every training or something that you get … that …uhmmm … um … um … it makes you more … um … um … more aware of things, not just things that fit into your field of work, but to learn how to look after yourself, how to handle things, so I grab every opportunity that I get.’ (Participant 12, female, 56 years old)

## Discussion

The aim of the study was twofold: (1) to explore the challenges CHWs’ experience in carrying out their duties and (2) to explore their motivation to join a self-management programme. Based on the findings of the study the emerging themes that describe the challenges are discussed initially and then the motivation to join the self-management programme.

### Social conditions and mental health

In this study, the CHWs reported that the social conditions of the patients led to fear, depression and an inability to focus on what the CHWs wanted them as patients to do. Literature from India commented on the CHWs’ description of not gaining access to clients’ homes as one of the negative experiences of the job.^35^ A recent study determined that social conditions do have an impact on the mental health of an individual.^36^ Although there is evidence that CHWs are not always safe when they enter the homes of patients with mental health problems,^37^ concern for safety was not raised in this study. Rather, CHWs observed the need to understand the patient and how to communicate depending on the mental health status of the patient, and CHWs in this study demonstrated empathy towards their clients’ mental health needs. Understanding the mental health conditions of patients thus becomes a key aspect of the skills that CHWs require. The need to include mental healthcare training and coordination at the primary care level is consistent with needs expressed by CHWs in this study.^38^ Using CHWs resourcefully in the primary care setting may result in stress reduction and mental health promotion amongst community patients, especially in low- and middle-income countries that are severely under-resourced with respect to mental health services.

### Patient adherence and communication

The lack of patient adherence to the prescribed treatment regimens were identified as a challenge. Factors contributing to the lack of adherence to proposed health regimens are multi-faceted, ranging from undervaluing preventative measures to psychosocial and socioeconomic hurdles to fears over stigma and challenges with traversing the health sector.^39,40,41^ As CHWs can play a key role in areas where health service access or motivation is poor,^13^ ‘training on behaviour change techniques’ would stand CHWs in good stead to deal with adherence concerns.^42^ Community health workers have themselves reported that they are capable of motivating community members to seek appropriate health services.^39^ It is thus apparent that CHWs can make a positive difference in assisting patients to adhere to medical regimens if they receive the correct training. Communication between the community and the health system is vital. The CHW workforce is like an important conveyor belt that transports the key health messages to the community and simultaneously increases the formal health professional’s awareness of the social determinants contributing to the patients’ health status. Community health workers in their dual role are more effective when they receive the respect they deserve from the formal health professionals and the community they serve because they feel that their contribution is valued.^43^ One advantage of the CHW workforce is that they come from the communities they serve and therefore have a unique ability to speak the language of the community.^40^ Although the community may not always understand the role of the CHW, it is important that communication channels remain open as it has been shown that CHWs are effective in strengthening communication between the medical system and the community.^44,45^

### Work environment and ineffective self-management

In this study the CHWs raised workload and the environment as a barrier to successfully implementing their duties. Literature has previously highlighted CHW workload as a barrier to achieving goals set by supervisors or health systems.^46^ In a study aptly titled ‘We are the people whose opinion don’t matter’, CHWs expressed their need to have their work environment challenges addressed by the NGOs with which they are affiliated.^47^ World-wide it has been reported that increased workload and the absence of clearly defined boundaries for the job causes stress.^48^ This is no different for CHWs, who are under tremendous pressure to meet health needs with subpar human resources, increasingly having additional responsibilities added to their workload and disconnection between themselves and the formal health sector.^47,49,50^ Studies have documented these factors as significant contributors to occupational stress.^51,52^

Aligned to the fact that the CHWs were not coping with their increased workloads, was their inability to successfully manage their own health. Literature reveals much research about the roles that CHWs play in addressing health challenges in the communities, yet there is a dearth of information on CHWs’ health needs and how they manage their own health. A substantial proportion of health professionals including CHWs struggle to manage their own health behaviours and to ‘practice what they preach’.^18,53^ A recent study amongst rural CHWs reported that they present with chronic conditions such as those of the community members they serve and that they also experience physical and emotional barriers to managing their own chronic conditions, suggesting that CHWs themselves may be good candidates for self-management interventions.^54^

### Motivations to participate in a self-management programme

In this study the participants highlighted empowerment and widening perspectives as two key reasons for joining a self-management programme. Research indicates that CHWs feel empowered when they are valued for the healthcare contributions they make, when they are included as part of the healthcare team and when they receive training to improve their competence.^35,43,55^ With the expectation that CHWs need to contribute productively to improvements in overall community health, it is important that CHWs feel empowered.^55^ Training is highly regarded amongst CHWs globally and a substantial contributor to CHW effectiveness and motivation. As such, CHWs need to be empowered to improve their own health behaviours and then to serve as a catalyst and role model by empowering the community members with increased knowledge and support.^56^ The role that CHWs play in communities has been flagged as an empowerment strategy to reach communities with the aim of improving healthcare. Once communities are empowered there is an increase in the sense of self-determination and self-efficacy and a positive cycle of health behaviours may be perpetuated and sustained.^57^

It has been found that CHWs were very keen to gain new knowledge, as such new information modified their worldview.^35^ This study echoed these findings, but it also yielded further reasons for the CHWs participation in the self-management programme. These included the enjoyment they receive from gaining new knowledge, possible job advancement, the transference of new-found knowledge to benefit others, learning coping skills and the desire to learn ways to manage their health. The participants in this study also indicated that they would not pass up an opportunity for training.

### Implications for practice

Research has shown that most engagement with the CHW workforce has been around upskilling to improve the health and empowerment in the communities they serve.^58^ Much has been documented about CHWs as a liaison between the formal healthcare system^13^ and the community and as a vital strategic resource to achieve global health goals, the actual CHWs and their own needs have been grossly overlooked. There is sufficient evidence to inform decision-makers about the factors that motivate CHWs into performing their roles effectively.^35,43^ Empowerment of this cadre should be high on the agenda, as CHWs cannot transfer skill sets of confidence, goal setting and action planning needed to create behaviour change without themselves developing and maintaining these skills. A self-management programme, which by its design is problem-based and incorporates the person’s perception in the process, is ideally suited as a tool to achieve this end, and CHWs as frontline providers are ideal candidates to be trained as self-managers.^20^ As members from the same communities they work in and share the same health and social needs as their communities, thus becoming effective self-managers will enhance CHWs’ cultural competence. Self-management programmes train participants in the key skills of decision-making, finding and utilising resources, forming partnerships with their healthcare providers, and taking action. The mastery of such skills would benefit CHWs in meeting role expectations.^21,59^

Simply belonging to the same community and having shared experiences is not sufficient to equip CHWs to work with clients who are unresponsive, who undervalue CHWs’ role and who do not adhere to recommended health behaviours. Therefore, there is a need to incorporate these competencies into all training offered to CHWs coupled with regular maintenance sessions. Self-management is a cost-effective strategy^60^ that can be used to address this, and it can therefore be successfully rolled out in low-income areas. As the CHWs become effective self-managers they can train community members and the positive cycle can continue.

The formal health system has been remiss in failing to fully accept the CHW as a critical link in the patient-centred delivery model.^45,47^ It is important that health systems develop insight into the critical role of CHWs, the impact CHWs make in their communities and work to remedy the strained relationship that currently exists between CHWs and health systems. Self-management is an empowerment strategy of meta-skills that does not rely on deep content knowledge within an aspect of medicine. One strategy to improve mutual respect could be to enable CHWs to serve as self-management trainers to members of the formal health system. During these trainings CHWs can present their field findings to formal health professionals. Community health workers-led training may improve the structural competence of health professionals. Another way to address this current divide is to have more ‘multidisciplinary’ engagements, wherein all parties have an equal voice to contribute.

## Conclusion

Community health workers affiliated with community-based organisations are central to the implementation of primary healthcare in district health services in South Africa. The themes presented here offer insight into the benefits and challenges described by CHWs. Although these findings are context-specific and so cannot be generalised to the global population, there is sufficient commonality amongst CHW roles worldwide to warrant an urgent response to these challenges. Hearing these often ‘silenced voices’ and responding with tangible risk mitigation strategies will support motivation and empowerment levels required by CHWs to work optimally. This study highlights that CHWs are eager to find solutions to these challenges and one of the ways they did it was by taking the opportunity to learn self-management skills by signing up for self-management training.

Equipping CHWs with self-management tools should positively influence the communities they serve and may ultimately result in healthier communities and a decreased disease burden.
